# 2-{(1*S*,2*S*,4a*R*,8*R*,8a*R*)-8-Hy­droxy-4a,8-dimethyl-1-[(2*E*)-2-methyl­but-2-eno­yl­oxy]perhydro­naphthalen-2-yl}acrylic acid from *Sclerorhachis platyrachis*

**DOI:** 10.1107/S1600536811037640

**Published:** 2011-09-30

**Authors:** Rasool Kheyrabadi, Zohreh Habibi, Seik Weng Ng

**Affiliations:** a Department of Chemistry, Shahid Beheshti University, General Campus, Tehran, Iran; b Department of Chemistry, University of Malaya, 50603 Kuala Lumpur, Malaysia; c Chemistry Department, Faculty of Science, King Abdulaziz University, PO Box 80203 Jeddah, Saudi Arabia

## Abstract

The eudesmane-type terpenoid, C_20_H_30_O_5_, isolated from *Sclerorhachis platyrachis*, has a deca­lin skeleton whose six-membered rings adopt chair conformations. The two methyl substituents occupy axial positions, whereas the other three substituents occupy equatorial positions. The hy­droxy group is an intra­molecular hydrogen-bond donor to the single-bond ester O atom; adjacent mol­ecules are linked through the carb­oxy­lic acid interacting with the hydroxyl group, forming a hydrogen-bonded chain running along the *c* axis.

## Related literature

For the crystal structure of epiilic acid, see: Daniewski *et al.* (1986 ▶). For a review of eudesmane-type sesquiterpenoids, see: Wu *et al.* (2006 ▶).
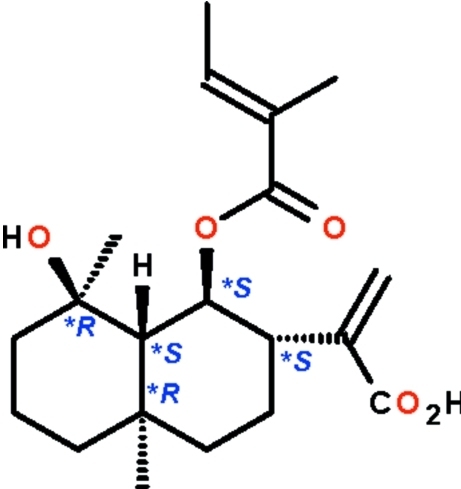


## Experimental

### 

#### Crystal data

C_20_H_30_O_5_
*M* *_r_* = 350.44
Monoclinic, 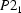

*a* = 6.2718 (1) Å
*b* = 19.0285 (3) Å
*c* = 8.4530 (2) Å
β = 110.184 (2)°
*V* = 946.85 (3) Å^3^
*Z* = 2
Cu *K*α radiation
μ = 0.71 mm^−1^
*T* = 100 K
0.20 × 0.20 × 0.20 mm

#### Data collection

Agilent SuperNova Dual diffractometer with an Atlas detector
Absorption correction: multi-scan (*CrysAlis PRO*; Agilent, 2010 ▶) *T*_min_ = 0.872, *T*_max_ = 0.872
5962 measured reflections
3663 independent reflections
3635 reflections with *I* > 2σ(*I*)
*R*_int_ = 0.015

#### Refinement

*R*[*F* ^2^ > 2σ(*F* ^2^)] = 0.028
*wR*(*F* ^2^) = 0.076
*S* = 1.03
3663 reflections
238 parameters
1 restraint
H atoms treated by a mixture of independent and constrained refinement
Δρ_max_ = 0.19 e Å^−3^
Δρ_min_ = −0.13 e Å^−3^
Absolute structure: Flack (1983 ▶), 1705 Friedel pairs
Flack parameter: 0.08 (11)

### 

Data collection: *CrysAlis PRO* (Agilent, 2010 ▶); cell refinement: *CrysAlis PRO*; data reduction: *CrysAlis PRO*; program(s) used to solve structure: *SHELXS97* (Sheldrick, 2008 ▶); program(s) used to refine structure: *SHELXL97* (Sheldrick, 2008 ▶); molecular graphics: *X-SEED* (Barbour, 2001 ▶); software used to prepare material for publication: *publCIF* (Westrip, 2010 ▶).

## Supplementary Material

Crystal structure: contains datablock(s) global, I. DOI: 10.1107/S1600536811037640/jh2330sup1.cif

Structure factors: contains datablock(s) I. DOI: 10.1107/S1600536811037640/jh2330Isup2.hkl

Supplementary material file. DOI: 10.1107/S1600536811037640/jh2330Isup3.cml

Additional supplementary materials:  crystallographic information; 3D view; checkCIF report

## Figures and Tables

**Table 1:** Hydrogen-bond geometry (Å, °)

*D*—H⋯*A*	*D*—H	H⋯*A*	*D*⋯*A*	*D*—H⋯*A*
O1—H1⋯O5^i^	0.85 (2)	1.80 (2)	2.648 (1)	174 (2)
O5—H5⋯O3	0.85 (2)	1.99 (2)	2.692 (1)	139 (2)

Symmetry code: (i) 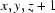.

**Fig. 1.**
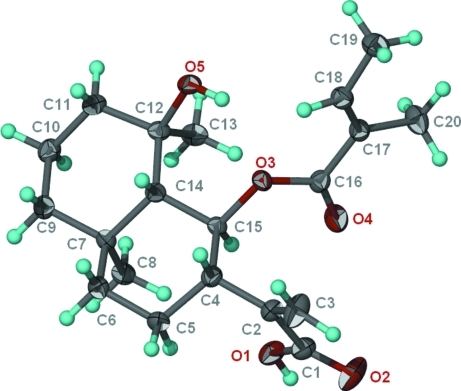
Thermal ellipsoid plot (Barbour, 2001) of C20H30O5 at the 70% probability level; hydrogen atoms are drawn as spheres of arbitrary radius.

**Fig. 2.**
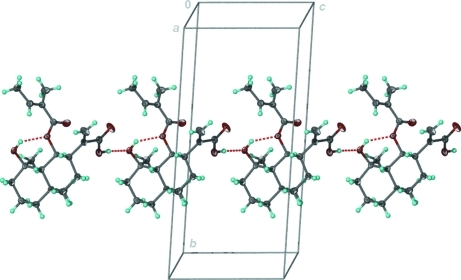
Hydrogen-bonded chain structure.

## supplementary crystallographic information

### Comment

Eudesmane-type of sesquiterpenoids (from the Asteraceae family) have been reviewed; some structural assignments and stereochemistries have also revised; however, the title compound (Scheme I) is not included in the review (Wu *et al.*, 2006). The compound is a derivative of epiilic (vachanic) acid having a 1-(2-methylbut-2-enoyloxy) substitutent. The crystal structure of the parent acid itself, which was first isolated from *Dittrichia vicossa* (*L*.), has been reported (Daniewski *et al.*, 186). The two methyl substituents occupy axial positions whereas the other three substituents occupy equatorial positions (Fig 1). The hydroxy group is an intramolecular hydrogen-bond donor to the single-bond ester O atom; adjacent molecules are linked through the carboxylic acid portion to form a hydrogen-bonded chain running along the *c*-axis of the monoclinic unit cell (Table 1).

### Experimental

The leaves and stems of *Sclerorhachis platyrachis* (Compositae family) were collected from Sabzevar, Khorasan Razavi Province, Iran, at the flowering stage of the plant, *i.e.*, around May. The aerial parts were dried in the shade. The aerial parts (300 g) were extracted with chloroform by maceration at room temperature. The extract was concentrated to a green gummy extract (16 g). The extract was subjected to column chromatography on silica gel (4×70 cm, 70–230 mesh) with a gradient of *n*-hexane–ethyl acetate and then methanol as eluent. Eighty-three fractions were collected according to TLC analysis and those giving similar spots were combined. Fraction 51 (*n*-hexane: ethylacetate 4:1) afforded colorless crystals of the title compound (500 mg).

### Refinement

Carbon-bound H-atoms were placed in calculated positions [C—H 0.95 to 0.98 Å, *U*_iso_(H) 1.2 to 1.5*U*_eq_(C)] and were included in the refinement in the riding model approximation.

The carboxylic and hydroxy H-atoms were located in a difference Fourier map, and were freely refined.

### Crystal data

**Table:**

C_20_H_30_O_5_	*F*(000) = 380
*M**_r_* = 350.44	*D*_x_ = 1.229 Mg m^−^^3^
Monoclinic, *P*2_1_	Cu *K*α radiation, λ = 1.54184 Å
Hall symbol: P 2yb	Cell parameters from 4968 reflections
*a* = 6.2718 (1) Å	θ = 5.6–74.1°
*b* = 19.0285 (3) Å	µ = 0.71 mm^−^^1^
*c* = 8.4530 (2) Å	*T* = 100 K
β = 110.184 (2)°	Prism, colourless
*V* = 946.85 (3) Å^3^	0.20 × 0.20 × 0.20 mm
*Z* = 2	

### Data collection

**Table:**

Agilent SuperNova Dual diffractometer with an Atlas detector	3663 independent reflections
Radiation source: SuperNova (Cu) X-ray Source	3635 reflections with *I* > 2σ(*I*)
Mirror	*R*_int_ = 0.015
Detector resolution: 10.4041 pixels mm^-1^	θ_max_ = 74.2°, θ_min_ = 5.6°
ω scans	*h* = −6→7
Absorption correction: multi-scan (*CrysAlis PRO*; Agilent, 2010)	*k* = −23→22
*T*_min_ = 0.872, *T*_max_ = 0.872	*l* = −10→10
5962 measured reflections	

### Refinement

**Table:**

Refinement on *F*^2^	Secondary atom site location: difference Fourier map
Least-squares matrix: full	Hydrogen site location: inferred from neighbouring sites
*R*[*F*^2^ > 2σ(*F*^2^)] = 0.028	H atoms treated by a mixture of independent and constrained refinement
*wR*(*F*^2^) = 0.076	*w* = 1/[σ^2^(*F*_o_^2^) + (0.0493*P*)^2^ + 0.1079*P*] where *P* = (*F*_o_^2^ + 2*F*_c_^2^)/3
*S* = 1.03	(Δ/σ)_max_ = 0.001
3663 reflections	Δρ_max_ = 0.19 e Å^−^^3^
238 parameters	Δρ_min_ = −0.13 e Å^−^^3^
1 restraint	Absolute structure: Flack (1983), 1705 Friedel pairs
Primary atom site location: structure-invariant direct methods	Flack parameter: 0.08 (11)

### Fractional atomic coordinates and isotropic or equivalent isotropic displacement parameters (Å^2^)

**Table:**

	*x*	*y*	*z*	*U*_iso_*/*U*_eq_	
O1	0.94364 (17)	0.50094 (5)	0.32350 (12)	0.0229 (2)	
O2	1.0317 (2)	0.39226 (6)	0.42340 (14)	0.0337 (2)	
O3	0.88724 (14)	0.44178 (4)	−0.13924 (11)	0.01664 (18)	
O4	0.64080 (17)	0.41443 (5)	−0.00727 (13)	0.0264 (2)	
O5	0.76555 (15)	0.51072 (5)	−0.43525 (11)	0.01914 (19)	
C1	1.0458 (2)	0.43922 (7)	0.33077 (16)	0.0212 (3)	
C2	1.1838 (2)	0.43410 (7)	0.21712 (15)	0.0190 (2)	
C3	1.3138 (3)	0.37790 (8)	0.2342 (2)	0.0305 (3)	
H3A	1.3167	0.3431	0.3155	0.037*	
H3B	1.4042	0.3723	0.1651	0.037*	
C4	1.1868 (2)	0.49268 (6)	0.09608 (15)	0.0170 (2)	
H4	1.2901	0.4766	0.0358	0.020*	
C5	1.2922 (2)	0.56070 (7)	0.19001 (15)	0.0200 (3)	
H5A	1.2069	0.5752	0.2636	0.024*	
H5B	1.4512	0.5514	0.2626	0.024*	
C6	1.2884 (2)	0.62031 (7)	0.06860 (16)	0.0199 (3)	
H6A	1.3928	0.6086	0.0073	0.024*	
H6B	1.3454	0.6637	0.1342	0.024*	
C7	1.0501 (2)	0.63443 (6)	−0.06021 (15)	0.0167 (2)	
C8	0.8926 (2)	0.65941 (7)	0.03410 (16)	0.0205 (3)	
H8A	0.9591	0.7008	0.1024	0.031*	
H8B	0.8751	0.6217	0.1075	0.031*	
H8C	0.7435	0.6717	−0.0477	0.031*	
C9	1.0683 (2)	0.69343 (7)	−0.17927 (17)	0.0198 (3)	
H9A	1.1013	0.7383	−0.1162	0.024*	
H9B	1.1968	0.6832	−0.2182	0.024*	
C10	0.8522 (2)	0.70175 (7)	−0.33205 (16)	0.0205 (3)	
H10A	0.7238	0.7141	−0.2946	0.025*	
H10B	0.8720	0.7402	−0.4047	0.025*	
C11	0.8004 (2)	0.63328 (7)	−0.43153 (15)	0.0196 (3)	
H11A	0.9268	0.6226	−0.4726	0.023*	
H11B	0.6611	0.6396	−0.5313	0.023*	
C12	0.7668 (2)	0.57076 (7)	−0.32876 (14)	0.0165 (2)	
C13	0.5355 (2)	0.57294 (7)	−0.30647 (15)	0.0210 (3)	
H13A	0.5234	0.5336	−0.2353	0.031*	
H13B	0.4151	0.5693	−0.4169	0.031*	
H13C	0.5196	0.6173	−0.2529	0.031*	
C14	0.97307 (19)	0.56460 (6)	−0.16172 (14)	0.0150 (2)	
H14	1.1038	0.5505	−0.1968	0.018*	
C15	0.95518 (19)	0.50674 (6)	−0.04079 (14)	0.0144 (2)	
H15	0.8402	0.5198	0.0118	0.017*	
C16	0.7264 (2)	0.40082 (6)	−0.11125 (15)	0.0178 (2)	
C17	0.6621 (2)	0.33936 (6)	−0.22701 (15)	0.0172 (2)	
C18	0.7910 (2)	0.32087 (7)	−0.31761 (16)	0.0196 (3)	
H18	0.9227	0.3486	−0.3021	0.024*	
C19	0.7516 (2)	0.26166 (7)	−0.44010 (17)	0.0249 (3)	
H19A	0.8832	0.2301	−0.4053	0.037*	
H19B	0.7301	0.2804	−0.5526	0.037*	
H19C	0.6156	0.2356	−0.4429	0.037*	
C20	0.4449 (2)	0.30420 (7)	−0.23055 (18)	0.0249 (3)	
H20A	0.4210	0.2614	−0.2990	0.037*	
H20B	0.3170	0.3364	−0.2795	0.037*	
H20C	0.4556	0.2920	−0.1154	0.037*	
H1	0.881 (4)	0.5016 (12)	0.399 (3)	0.053 (6)*	
H5	0.787 (4)	0.4729 (12)	−0.379 (3)	0.041 (5)*	

### Atomic displacement parameters (Å^2^)

**Table:**

	*U*^11^	*U*^22^	*U*^33^	*U*^12^	*U*^13^	*U*^23^
O1	0.0310 (5)	0.0223 (5)	0.0201 (4)	0.0048 (4)	0.0150 (4)	0.0010 (4)
O2	0.0468 (6)	0.0291 (5)	0.0353 (6)	0.0089 (5)	0.0270 (5)	0.0130 (5)
O3	0.0187 (4)	0.0162 (4)	0.0172 (4)	−0.0033 (3)	0.0090 (3)	−0.0039 (3)
O4	0.0302 (5)	0.0262 (5)	0.0305 (5)	−0.0080 (4)	0.0205 (4)	−0.0073 (4)
O5	0.0248 (5)	0.0190 (5)	0.0144 (4)	0.0004 (4)	0.0078 (3)	−0.0025 (4)
C1	0.0245 (6)	0.0220 (6)	0.0178 (6)	0.0016 (5)	0.0082 (5)	0.0016 (5)
C2	0.0211 (5)	0.0192 (6)	0.0170 (5)	−0.0002 (5)	0.0069 (4)	0.0022 (5)
C3	0.0377 (8)	0.0240 (7)	0.0382 (8)	0.0084 (6)	0.0238 (7)	0.0095 (6)
C4	0.0175 (6)	0.0182 (6)	0.0162 (5)	−0.0002 (4)	0.0069 (5)	0.0005 (5)
C5	0.0199 (6)	0.0217 (7)	0.0153 (5)	−0.0035 (5)	0.0019 (4)	0.0003 (5)
C6	0.0185 (6)	0.0209 (6)	0.0194 (6)	−0.0042 (5)	0.0053 (5)	0.0000 (5)
C7	0.0186 (6)	0.0156 (6)	0.0159 (5)	−0.0025 (4)	0.0058 (5)	−0.0017 (4)
C8	0.0244 (6)	0.0189 (6)	0.0193 (6)	−0.0004 (5)	0.0090 (5)	−0.0041 (5)
C9	0.0218 (6)	0.0169 (6)	0.0214 (6)	−0.0017 (4)	0.0083 (5)	0.0003 (4)
C10	0.0252 (6)	0.0161 (6)	0.0207 (6)	0.0034 (5)	0.0084 (5)	0.0027 (5)
C11	0.0220 (6)	0.0212 (6)	0.0150 (6)	0.0017 (5)	0.0057 (5)	0.0010 (5)
C12	0.0184 (6)	0.0170 (6)	0.0147 (5)	0.0011 (5)	0.0063 (4)	−0.0024 (5)
C13	0.0166 (6)	0.0269 (7)	0.0189 (6)	0.0014 (5)	0.0054 (5)	−0.0025 (5)
C14	0.0147 (5)	0.0168 (6)	0.0147 (5)	−0.0004 (4)	0.0067 (4)	−0.0020 (5)
C15	0.0167 (6)	0.0133 (5)	0.0141 (5)	−0.0008 (4)	0.0065 (4)	−0.0027 (4)
C16	0.0161 (5)	0.0196 (6)	0.0186 (6)	−0.0007 (4)	0.0072 (4)	0.0017 (5)
C17	0.0166 (5)	0.0157 (6)	0.0182 (5)	−0.0002 (4)	0.0045 (4)	0.0013 (4)
C18	0.0186 (6)	0.0196 (6)	0.0197 (6)	−0.0010 (5)	0.0054 (5)	−0.0006 (5)
C19	0.0265 (6)	0.0251 (7)	0.0232 (7)	−0.0012 (5)	0.0088 (5)	−0.0056 (5)
C20	0.0199 (6)	0.0232 (7)	0.0333 (7)	−0.0044 (5)	0.0113 (5)	−0.0041 (5)

### Geometric parameters (Å, °)

**Table:**

O1—C1	1.3290 (16)	C9—C10	1.5236 (18)
O1—H1	0.85 (2)	C9—H9A	0.9900
O2—C1	1.2113 (16)	C9—H9B	0.9900
O3—C16	1.3586 (15)	C10—C11	1.5237 (18)
O3—C15	1.4689 (13)	C10—H10A	0.9900
O4—C16	1.2056 (16)	C10—H10B	0.9900
O5—C12	1.4530 (14)	C11—C12	1.5297 (17)
O5—H5	0.85 (2)	C11—H11A	0.9900
C1—C2	1.5021 (17)	C11—H11B	0.9900
C2—C3	1.322 (2)	C12—C13	1.5265 (16)
C2—C4	1.5175 (16)	C12—C14	1.5559 (15)
C3—H3A	0.9500	C13—H13A	0.9800
C3—H3B	0.9500	C13—H13B	0.9800
C4—C15	1.5365 (15)	C13—H13C	0.9800
C4—C5	1.5429 (17)	C14—C15	1.5325 (16)
C4—H4	1.0000	C14—H14	1.0000
C5—C6	1.5245 (17)	C15—H15	1.0000
C5—H5A	0.9900	C16—C17	1.4885 (16)
C5—H5B	0.9900	C17—C18	1.3388 (18)
C6—C7	1.5394 (17)	C17—C20	1.5083 (17)
C6—H6A	0.9900	C18—C19	1.4920 (17)
C6—H6B	0.9900	C18—H18	0.9500
C7—C9	1.5380 (17)	C19—H19A	0.9800
C7—C8	1.5428 (17)	C19—H19B	0.9800
C7—C14	1.5650 (16)	C19—H19C	0.9800
C8—H8A	0.9800	C20—H20A	0.9800
C8—H8B	0.9800	C20—H20B	0.9800
C8—H8C	0.9800	C20—H20C	0.9800
			
C1—O1—H1	108.4 (15)	H10A—C10—H10B	108.2
C16—O3—C15	118.15 (9)	C10—C11—C12	113.36 (10)
C12—O5—H5	110.5 (14)	C10—C11—H11A	108.9
O2—C1—O1	122.71 (12)	C12—C11—H11A	108.9
O2—C1—C2	123.53 (12)	C10—C11—H11B	108.9
O1—C1—C2	113.74 (11)	C12—C11—H11B	108.9
C3—C2—C1	116.90 (12)	H11A—C11—H11B	107.7
C3—C2—C4	121.14 (12)	O5—C12—C13	107.20 (10)
C1—C2—C4	121.84 (11)	O5—C12—C11	103.42 (9)
C2—C3—H3A	120.0	C13—C12—C11	111.83 (11)
C2—C3—H3B	120.0	O5—C12—C14	109.15 (9)
H3A—C3—H3B	120.0	C13—C12—C14	114.71 (9)
C2—C4—C15	114.03 (10)	C11—C12—C14	109.86 (10)
C2—C4—C5	111.84 (10)	C12—C13—H13A	109.5
C15—C4—C5	111.54 (10)	C12—C13—H13B	109.5
C2—C4—H4	106.3	H13A—C13—H13B	109.5
C15—C4—H4	106.3	C12—C13—H13C	109.5
C5—C4—H4	106.3	H13A—C13—H13C	109.5
C6—C5—C4	111.93 (10)	H13B—C13—H13C	109.5
C6—C5—H5A	109.2	C15—C14—C12	115.48 (9)
C4—C5—H5A	109.2	C15—C14—C7	108.91 (9)
C6—C5—H5B	109.2	C12—C14—C7	115.86 (10)
C4—C5—H5B	109.2	C15—C14—H14	105.1
H5A—C5—H5B	107.9	C12—C14—H14	105.1
C5—C6—C7	113.06 (10)	C7—C14—H14	105.1
C5—C6—H6A	109.0	O3—C15—C14	107.48 (8)
C7—C6—H6A	109.0	O3—C15—C4	107.10 (9)
C5—C6—H6B	109.0	C14—C15—C4	111.08 (9)
C7—C6—H6B	109.0	O3—C15—H15	110.4
H6A—C6—H6B	107.8	C14—C15—H15	110.4
C6—C7—C9	108.48 (10)	C4—C15—H15	110.4
C6—C7—C8	109.08 (10)	O4—C16—O3	123.23 (11)
C9—C7—C8	108.57 (10)	O4—C16—C17	124.06 (11)
C6—C7—C14	106.28 (10)	O3—C16—C17	112.67 (10)
C9—C7—C14	109.85 (9)	C18—C17—C16	120.16 (11)
C8—C7—C14	114.42 (10)	C18—C17—C20	126.28 (12)
C7—C8—H8A	109.5	C16—C17—C20	113.55 (11)
C7—C8—H8B	109.5	C17—C18—C19	127.40 (12)
H8A—C8—H8B	109.5	C17—C18—H18	116.3
C7—C8—H8C	109.5	C19—C18—H18	116.3
H8A—C8—H8C	109.5	C18—C19—H19A	109.5
H8B—C8—H8C	109.5	C18—C19—H19B	109.5
C10—C9—C7	112.67 (10)	H19A—C19—H19B	109.5
C10—C9—H9A	109.1	C18—C19—H19C	109.5
C7—C9—H9A	109.1	H19A—C19—H19C	109.5
C10—C9—H9B	109.1	H19B—C19—H19C	109.5
C7—C9—H9B	109.1	C17—C20—H20A	109.5
H9A—C9—H9B	107.8	C17—C20—H20B	109.5
C11—C10—C9	109.70 (10)	H20A—C20—H20B	109.5
C11—C10—H10A	109.7	C17—C20—H20C	109.5
C9—C10—H10A	109.7	H20A—C20—H20C	109.5
C11—C10—H10B	109.7	H20B—C20—H20C	109.5
C9—C10—H10B	109.7		
			
O2—C1—C2—C3	−7.2 (2)	C13—C12—C14—C7	−79.68 (14)
O1—C1—C2—C3	171.30 (13)	C11—C12—C14—C7	47.30 (13)
O2—C1—C2—C4	176.65 (13)	C6—C7—C14—C15	63.08 (12)
O1—C1—C2—C4	−4.81 (17)	C9—C7—C14—C15	−179.76 (9)
C3—C2—C4—C15	120.75 (14)	C8—C7—C14—C15	−57.36 (13)
C1—C2—C4—C15	−63.31 (15)	C6—C7—C14—C12	−164.75 (10)
C3—C2—C4—C5	−111.53 (15)	C9—C7—C14—C12	−47.59 (13)
C1—C2—C4—C5	64.41 (15)	C8—C7—C14—C12	74.81 (13)
C2—C4—C5—C6	−178.17 (10)	C16—O3—C15—C14	−137.12 (10)
C15—C4—C5—C6	−49.13 (14)	C16—O3—C15—C4	103.46 (11)
C4—C5—C6—C7	53.90 (14)	C12—C14—C15—O3	48.75 (12)
C5—C6—C7—C9	−178.02 (10)	C7—C14—C15—O3	−178.88 (8)
C5—C6—C7—C8	63.89 (13)	C12—C14—C15—C4	165.60 (9)
C5—C6—C7—C14	−59.95 (13)	C7—C14—C15—C4	−62.02 (12)
C6—C7—C9—C10	168.69 (10)	C2—C4—C15—O3	−60.90 (12)
C8—C7—C9—C10	−72.89 (13)	C5—C4—C15—O3	171.23 (9)
C14—C7—C9—C10	52.92 (13)	C2—C4—C15—C14	−177.99 (10)
C7—C9—C10—C11	−59.35 (14)	C5—C4—C15—C14	54.14 (12)
C9—C10—C11—C12	59.45 (14)	C15—O3—C16—O4	−1.68 (17)
C10—C11—C12—O5	−169.09 (10)	C15—O3—C16—C17	176.10 (9)
C10—C11—C12—C13	75.89 (13)	O4—C16—C17—C18	−168.19 (13)
C10—C11—C12—C14	−52.68 (13)	O3—C16—C17—C18	14.06 (16)
O5—C12—C14—C15	−70.92 (12)	O4—C16—C17—C20	12.88 (18)
C13—C12—C14—C15	49.37 (14)	O3—C16—C17—C20	−164.88 (11)
C11—C12—C14—C15	176.34 (9)	C16—C17—C18—C19	−179.39 (12)
O5—C12—C14—C7	160.04 (9)	C20—C17—C18—C19	−0.6 (2)

### Hydrogen-bond geometry (Å, °)

**Table:**

*D*—H···*A*	*D*—H	H···*A*	*D*···*A*	*D*—H···*A*
O1—H1···O5^i^	0.85 (2)	1.80 (2)	2.648 (1)	174 (2)
O5—H5···O3	0.85 (2)	1.99 (2)	2.692 (1)	139 (2)

Symmetry codes: (i) *x*, *y*, *z*+1.
